# The role of exercise-induced short-chain fatty acids in the gut–muscle axis: implications for sarcopenia prevention and therapy

**DOI:** 10.3389/fmicb.2025.1665551

**Published:** 2025-10-08

**Authors:** Junyi Fang, Weiyi Yan, Xuao Sun, Jun Chen

**Affiliations:** ^1^School of Graduate, Wuhan Sports University, Wuhan, China; ^2^Guangming Liyuan School Affiliated to Sustech, Shenzhen, China; ^3^School of Art, Wuhan Sports University, Wuhan, China

**Keywords:** sarcopenia, short-chain fatty acids, exercise, gut microbiota, gut-muscle axis

## Abstract

Sarcopenia is an age-related syndrome characterized by a progressive loss of skeletal muscle mass and function, with its prevalence increasing annually and severely compromising the quality of life in older adults. The pathogenesis of sarcopenia is complex and closely associated with gut microbiota dysbiosis. Emerging evidence suggests that short-chain fatty acids (SCFAs), the main metabolites produced by the gut microbiota, act as key mediators linking gut microbes to skeletal muscle health, a relationship referred to as the gut–muscle axis. SCFAs not only regulate muscle protein metabolism and inflammatory responses but also improve skeletal muscle insulin sensitivity and mitochondrial function, thereby playing a crucial role in maintaining muscle health. Notably, exercise has been shown to increase the abundance of SCFA-producing bacteria in the gut of older adults, thereby elevating circulating SCFA levels. This review summarizes the effects of different exercise modalities on SCFA-producing gut microbiota and circulating SCFA levels in older adults. Furthermore, it discusses the potential mechanisms through which exercise-induced SCFAs contribute to the prevention and management of age-related sarcopenia, thereby providing new insights and scientific references for exercise-based strategies to prevent and treat this condition.

## 1 Introduction

Sarcopenia is an age-related degenerative condition characterized by a progressive decline in skeletal muscle mass, strength, and function, and it has become a growing global public health concern. Epidemiological data indicate that the prevalence of sarcopenia among older adults ranges from approximately 10–27% and increasing markedly with age (Petermann-Rocha et al., 2022). By 2050, the global number of individuals affected by sarcopenia is projected to exceed 200 million (Almohaisen et al., 2022). Importantly, sarcopenia is significantly associated with an increased risk of falls, physical disability, cognitive decline, and mortality in both healthy older adults and those with chronic diseases (Coletta and Phillips, 2023). These adverse outcomes lead to greater healthcare utilization and impose a considerable economic burden on families and society. Therefore, identifying effective therapeutic targets and preventive strategies is essential for delaying or reversing the progression of sarcopenia.

The pathogenesis of sarcopenia is multifactorial and remains incompletely understood. Current evidence indicates that it results from a complex interplay among anabolic resistance, chronic low-grade inflammation, mitochondrial dysfunction, and insulin resistance (Grima-Terrén et al., 2024; Bowen et al., 2015). In recent years, growing evidence has highlighted the critical role of gut microbiota dysbiosis in the onset and progression of sarcopenia (Tang et al., 2023; Zhang et al., 2023; Park et al., 2022). Aging is typically accompanied by reduced gut microbial diversity, increased harmful bacteria, and decreased beneficial taxa (Wang et al., 2024). These detrimental microbial changes may disrupt host metabolic homeostasis by promoting systemic inflammation, impairing muscle protein synthesis, and altering glucose metabolism, ultimately contributing to declines in muscle mass and function (Li et al., 2024).

Gut microbiota influence host metabolism and muscle health primarily through their metabolites. Among these, short-chain fatty acids (SCFAs)—the primary products of microbial fermentation of dietary fiber—are recognized as key mediators linking gut microbiota to skeletal muscle homeostasis (Liu et al., 2024; Li et al., 2024). It should be noted that the term “gut microbiota” broadly includes bacteria, archaea, viruses, and fungi; however, because SCFAs are predominantly generated by bacterial fermentation, the present review specifically focuses on the bacterial component of the gut microbiota. SCFAs serve not only as an essential energy source for colonic epithelial cells but also play crucial roles in modulating immune and inflammatory responses, enhancing mitochondrial function and energy metabolism, and improving insulin sensitivity (Chen et al., 2024; Mann et al., 2024; Mukhopadhya and Louis, 2025). However, in older individuals, the abundance of SCFA-producing bacteria declines significantly, resulting in reduced SCFA levels, suggesting that SCFA deficiency may represent a potential mechanism underlying the development of sarcopenia (Peng et al., 2023).

Exercise is a safe and effective non-pharmacological intervention that has been extensively shown to improve muscle mass, enhance muscle strength and function, and delay age-related muscle decline in older adults (Chen et al., 2024). Notably, exercise can beneficially modulate gut microbiota composition, increase the abundance of SCFA-producing bacteria, and consequently elevate systemic SCFA levels (Erlandson et al., 2021). These exercise-induced changes may promote skeletal muscle health via the gut–muscle axis, a bidirectional communication pathway linking gut microbiota and skeletal muscle function (Chen et al., 2021; Otsuka et al., 2023; Walsh et al., 2015). Therefore, this review focuses on the role of SCFAs in regulating the gut–muscle axis, explores the effects of exercise interventions on SCFA levels in older adults, and discusses the potential mechanisms by which exercise may prevent and treatment sarcopenia. The aim is to provide novel insights and a scientific basis for the prevention and management of sarcopenia through exercise-based strategies.

## 2 Overview of SCFAs

SCFAs are small-molecule carboxylic acids containing 1 to 6 carbon atoms, predominantly generated through the anaerobic fermentation of indigestible polysaccharides by gut microbiota. Among them, acetate, propionate, and butyrate are the major SCFAs, collectively accounting for approximately 95% of the total, with an average molar ratio of 3:1:1(Layden et al., 2013). Other SCFAs, such as formate, valerate, and caproate, are present in relatively lower concentrations. SCFA biosynthesis is taxonomically and metabolically distinct: acetate is mainly produced by *Bacteroides*, *Bifidobacterium*, and *Akkermansia* via the Wood–Ljungdahl pathway or through pyruvate oxidative decarboxylation (Ragsdale and Pierce, 2008); propionate is generated via the succinate pathway (typically by *Bacteroidetes*) or the lactate pathway (typically by *Firmicutes*) (Kircher et al., 2022); and butyrate is formed through the condensation of two acetyl-CoA molecules to butyryl-CoA, followed by conversion to butyrate via enzymes such as phosphotransbutyrylase or butyrate kinase, primarily by butyrate-producing bacteria including *Clostridium* and *Faecalibacterium (*Daskova et al., 2021*)*.

After diffusing into the cytoplasm of intestinal epithelial cells, butyrate serves as a key energy source via oxidative catabolism. The remaining approximately 5% of SCFAs rapidly dissociate into SCFA^−^ and H^+^ (Gäbel and Sehested, 1997). Apical transporters, including monocarboxylate transporter 1 (MCT1) and sodium-coupled monocarboxylate transporter 1 (SMCT1), mediate the uptake of SCFA^−^, Na^+^, and H^+^. To prevent intracellular acidification, sodium/hydrogen exchanger 1 (NHE1) exports excess H^+^ (Stumpff, 2018). Meanwhile, Na^+^ is extruded by the basolateral sodium-potassium pump, with K^+^ flux balanced through potassium channels. SCFA^−^ is also transported out via volume-regulated anion channels (VRAC) to maintain ionic homeostasis (Sivaprakasam et al., 2017).

SCFAs produced in the cecum, ascending colon, and transverse colon primarily enter the superior mesenteric vein, whereas those from the descending and sigmoid colon enter the inferior mesenteric vein; together, they reach the liver via the portal vein. In humans, most colonic SCFAs undergo first-pass utilization by colonocytes and the liver, markedly limiting systemic appearance—especially for propionate and butyrate. Colon-targeted isotope studies estimate systemic availability at ~36% (acetate), ~9% (propionate), and ~2% (butyrate); ~6% of colonic propionate enters glucose, while <1% of acetate and <15% into cholesterol and long-chain fatty acids (Boets et al., 2017). Portal–systemic sampling demonstrates a strong gradient from portal to hepatic to peripheral blood: peripheral venous propionate is typically 3–4 μM and butyrate is often near the detection limit, despite much higher portal concentrations (Peters et al., 1992). Consistent with these kinetics, peripheral SCFA concentrations are low but measurable (acetate predominates) and can increase after fermentable-fiber interventions in young and older adults, yet remain modest overall (Kirschner et al., 2025). Accordingly, while a fraction of SCFAs does enter the systemic circulation via the hepatic vein and may influence extra-intestinal physiology, mechanistic claims in peripheral tissues should be interpreted within this pharmacokinetic constraint.

Moreover, SCFAs act as signaling molecules by binding to G-protein coupled receptors (GPRs), such as GPR41, GPR43, and GPR109A, which are widely expressed across tissues (Koh et al., 2016). Notably, GPR41 and GPR43 are abundantly present on skeletal muscle cell membranes (Canfora et al., 2015). Studies in germ-free mice have demonstrated that exogenous SCFA supplementation (provided via drinking water and thus fully systemically available) can partially restore muscle mass and exercise capacity, underscoring the crucial role of SCFAs in maintaining skeletal muscle homeostasis (Lahiri et al., 2019). In humans, however, SCFAs are primarily produced in the colon and undergo extensive first-pass metabolism by colonocytes and the liver, resulting in a steep tissue availability gradient and relatively lower peripheral concentrations compared with animal models (Mukhopadhya and Louis, 2025). Nevertheless, even at relatively low systemic concentrations, SCFAs retain the ability to act as potent signaling molecules, modulating host metabolism, immune function, and skeletal muscle physiology (Mukhopadhya and Louis, 2025).

## 3 Age-related gut dysbiosis and reduced SCFA-producing bacteria in sarcopenia

With advancing age, the gut microbiota undergoes significant alterations, including decreased microbial diversity, reduced relative abundance of SCFA-producing bacteria, and increased abundance of pro-inflammatory and potentially pathogenic taxa. A cross-sectional study by Claesson et al. (2011) analyzed fecal samples from 161 older adults (≥65 years) and 9 younger adults (28–46 years), revealing a marked depletion of SCFA-producing bacteria in the elderly, particularly a pronounced reduction in the phylum *Firmicutes*. More than 65% of the older individuals exhibited an imbalanced *Firmicutes*-to-*Bacteroidetes* (F/B) ratio, a hallmark of microbial dysbiosis. Similar findings from other studies have shown that, compared to younger individuals, older adults exhibit reduced abundance of key SCFA-producing bacteria, such as *Lactobacillus* and *Bifidobacterium*, along with increased abundance of Gram-negative, endotoxin-producing, and pro-inflammatory bacteria (Lozada-Martinez et al., 2024; Biagi et al., 2016). These alterations may impair intestinal barrier function and lead to a persistent state of low-grade systemic inflammation (Chen et al., 2025). Animal models provide further support for these observations. Compared to young mice, aged mice exhibit significantly reduced fecal concentrations of SCFAs, including acetate, butyrate, isobutyrate, valerate, and isovalerate. Moreover, SCFA levels are positively correlated with the abundance of key SCFA-producing genera such as *Coprococcus*, *Ruminococcus*, and *Anaerorhabdus*, suggesting a potential interplay among aging, alterations in gut microbiota composition, and reduced SCFA production (Ma et al., 2025).

Additionally, accumulating evidence indicates that individuals with sarcopenia show a more profound reduction in SCFA-producing bacteria and SCFA concentrations compared to non-sarcopenic older adults. For example, Jiang et al. (2022) reported that older adults with sarcopenia exhibited significantly reduced gut microbial diversity and a marked decline in the abundance of *Marvinbryantia*, a known SCFA-producing genus, compared to non-sarcopenic older individuals. Notably, fecal butyrate concentrations were significantly lower in sarcopenic individuals and positively correlated with skeletal muscle mass index (Jiang et al., 2022). Similarly, Ticinesi et al. (2020) found that the fecal abundance of *Faecalibacterium prausnitzii*, *Roseburia inulinivorans*, and *Alistipes shahii*—bacteria known for their capacity to metabolize SCFAs—was significantly lower in sarcopenic individuals than in non-sarcopenic older controls. Further KEGG pathway analysis revealed downregulation of genes involved in SCFA synthesis and amino acid interconversion in the sarcopenic group. These studies suggest that SCFA deficiency and SCFA-related microbial dysbiosis are prevalent in the older adults and sarcopenia patients.

## 4 SCFAs: key players in the gut–muscle axis

The gut microbiota plays a vital role in several essential physiological processes, including food digestion and energy harvest, nutrient metabolism and absorption, immune regulation, and maintenance of gastrointestinal barrier integrity (Mukhopadhya and Louis, 2025). The composition of the gut microbiota and its metabolites is influenced by various intrinsic and extrinsic factors, such as diet, medication use, age, sex, and physical activity (Huang et al., 2024; Chen et al., 2021; Garcia-Santamarina et al., 2024). These microorganisms not only maintain local intestinal homeostasis but also mediate bidirectional communication with distant organs, including skeletal muscle, through their metabolic products, thereby contributing to systemic homeostasis (Li et al., 2022). In this context, the concept of the “gut–muscle axis” has emerged, highlighting the bidirectional regulatory relationship between gut microbial ecology and skeletal muscle health.

As previously discussed, SCFAs, the primary metabolites of gut microbiota, act as key mediators of the gut–muscle axis. A longitudinal study involving 823 community-dwelling older adults (≥60 years) showed that higher dietary SCFA intake was significantly associated with a lower risk of muscle strength decline over an average follow-up of 7.8 years (Otsuka et al., 2023). In an animal study, Zhu et al. (2024) reported that fecal microbiota transplantation (FMT) from young to aged mice significantly elevated serum SCFA levels, enhanced muscle mass and grip strength, and reduced systemic inflammation. These effects may be mediated by SCFAs through restoration of intestinal barrier integrity and inhibition of lipopolysaccharide (LPS) translocation (Zhu et al., 2024). Similarly, Lee et al. (2023) that supplementation with animal protein hydrolysates in aged mice restored total SCFA levels. The increased SCFA levels were positively correlated with muscle protein content and negatively associated with pro-inflammatory cytokine levels, suggesting that SCFAs may help prevent sarcopenia by enhancing protein synthesis and reducing inflammation (Lee et al., 2023). In another study, Liu et al. (2024) found that aged sarcopenic mice had lower serum butyrate levels, reduced muscle mass, and diminished strength compared to non-sarcopenic controls, all of which significantly improved after SCFA supplementation.

In addition to regulating inflammation and protein metabolism, SCFAs have been observed to improve mitochondrial function and enhance insulin sensitivity in skeletal muscle. For example, Walsh et al. (2015) demonstrated that butyrate supplementation in aged mice enhanced mitochondrial biogenesis in skeletal muscle, promoted hepatic gluconeogenesis, attenuated muscle atrophy, and decreased intramuscular fat accumulation. Additionally, treatment with propionate and valerate increased glucose uptake in 3 T3-L1 adipocytes and C2C12 myotubes, thereby enhancing insulin sensitivity (Han et al., 2014).

Collectively, these findings highlight the multifaceted roles of SCFAs in preserving muscle health among older adults. By modulating inflammation, promoting muscle protein synthesis, improving insulin sensitivity, and enhancing mitochondrial function, SCFAs represent promising targets for the prevention and treatment of age-related sarcopenia. However, it should be noted that much of the supporting evidence comes from animal and cell studies, making it difficult to directly extrapolate to older adults (e.g., oral butyrate supplementation may induce systemic effects that are unlikely to be achieved in humans). Moreover, in human studies, individuals with higher dietary SCFA intake also consumed greater amounts of protein and energy, both of which contribute to muscle strength. Therefore, future research should adopt more rigorous approaches, such as controlled dietary interventions and mechanistic studies, to clarify the independent role of SCFAs in older adults muscle health.

## 5 Effects of exercise on SCFA-producing bacteria and SCFA levels in older adults

Exercise acts as a powerful modulator of the gut microbiota, promoting the growth of SCFA-producing bacteria and thereby increasing SCFA production (Allen et al., 2018). In older adults, various exercise modalities—including aerobic, resistance, and combined exercise—have been extensively investigated as interventions to prevent and manage sarcopenia. This section summarizes and discusses the effects of these three primary exercise strategies on gut SCFA-producing bacteria and SCFA levels in older adults (Table 1).

**Table 1:** To summarize the effects of exercise on SCFA-producing bacteria in older aduls.

Object	Exercise intervention protocol	SCFA-producing bacteria	References
Healthy older women	Brisk walking, intensity of≥3 METs, 3 times/week, 60 min/session, 12 weeks	*Bacteroides*↑	Morita et al. (2019)
Healthy elderly men	Cycle ergometer, 55–77% VO_2max_, 3 times/week, 45 min/session, 5 weeks	*Oscillospira*↑	Taniguchi et al. (2018)
Middle-aged and elderly people	RE, 75% 1RM, 3 × 10 reps: leg press, leg curl, leg extension; two upper body exercises alternated, 3 times/week, 10 weeks	*Veillonellaceae* and *Akkermansia*↑	McKenna et al. (2021)
Healthy older adults	RE, RPE: 7–10, squats, 3 × 10–12 reps, 2 times/week, 10 weeks	Total SCFAs →	Agyin-Birikorang et al. (2025)
Sedentary older women	AE + RE, 4 times/week, 8 weeks AE: 20 min, stretching exercise; RE: six exercises of the upperand lower body using an elastic band, 3 × 12–15 reps	*Bifidobacteriaceae*、*Lachnospiraceae* and *Mitsuokella*↑	Zhong et al. (2021)
Obese adults	AE + RE, 3 times/week, 24 weeks AE: 50 min, 60–70% VO_2max_; RE: 60–80% 1RM, bench press, leg press, side pull, rotation, 3 × 8 reps	Oscillospira、Anaerostipes and fecal butyrate↑	Erlandson et al. (2021)

↑: up-regulation; ↓: down-regulation; →: no significant change; SCFAs: short chain fatty acids; METs: metabolic equivalants; RPE: rating of perceived exertion; VO_2max_: maximum oxygen uptake; AE: aerobic exercise; RE: resistance exercise; 1RM: one repetition maximum; reps: repetitions.

### 5.1 Aerobic exercise

Aerobic exercise can positively modulate the gut microbiota composition, particularly increasing abundance of SCFA-producing bacteria in in older adults. In a large-scale observational study involving 897 older adults aged over 60 years, Zhu et al. (2020) reported that, compared to sedentary individuals, those who regularly engaged in aerobic physical activity exhibited significantly higher relative abundances of *Firmicutes* and *Verrucomicrobia* at the phylum level, and *Prevotellaceae* and *Verrucomicrobiaceae* at the family level. These microbiota shifts provide a potential foundation for increased SCFA production. Interestingly, the effects were particularly pronounced in overweight older adults, suggesting that exercise-induced remodeling of the gut microbiota may be especially beneficial for metabolically impaired populations (Zhu et al., 2020). Morita et al. (2019) found that a 12-week aerobic training intervention in healthy older women significantly increased the relative abundance of *Bacteroides*. In a shorter-term study, Taniguchi et al. (2018) demonstrated that only 5 weeks of moderate-intensity aerobic training significantly increased the fecal abundance of Oscillospira, a known butyrate-producing genus, in older men.

Additionally, some studies have shown that the abundance of SCFA-producing bacteria, such as *Bifidobacterium* and *Lactobacillus*, increases with the duration of exercise intervention, indicating a possible time–dose relationship (Hamasaki, 2017; Yang et al., 2021; Bonomini-Gnutzmann et al., 2022). However, it is important to note that excessive or high-intensity exercise may adversely affect the gut microbiota. Overtraining has been associated with a reduction in SCFA-producing bacteria, increased gut permeability, and disruption of mucosal immune homeostasis, potentially triggering systemic inflammation (Yuan et al., 2018). Therefore, in older individuals, moderate-intensity aerobic exercise—such as brisk walking, cycling, or swimming at 50–70% of maximal heart rate for about 30–60 min per session, 3–5 times per week—is generally recommended to optimize gut microbial balance and promote SCFA production while minimizing potential adverse effects (Izquierdo et al., 2021).

Although several studies have demonstrated that aerobic exercise increases the abundance of intestinal SCFA-producing bacteria in older adults, few have directly measured SCFA concentrations in fecal or serum samples. Nonetheless, emerging evidence from both human and animal studies suggests that aerobic exercise may enhance SCFA production by modulating the gut microbiota. For example, Torquati et al. (2023) investigated the effects of an 8-week moderate-intensity aerobic exercise intervention in patients with type 2 diabetes mellitus and reported increased relative abundances of SCFA-producing bacteria such as *Bifidobacterium* and *Akkermansia muciniphila*, accompanied by elevated fecal SCFA levels, including acetate, propionate, and butyrate. Consistent with these findings, Matsumoto et al. (2008) showed that 4 weeks of voluntary treadmill running significantly increased cecal butyrate concentrations in rats. These results support the notion that moderate aerobic exercise can promote SCFA production by reshaping gut microbial composition. However, whether similar effects occur in older adults remains unclear. Therefore, further well-designed randomized controlled trials are warranted to elucidate whether aerobic exercise promotes SCFA synthesis through gut microbiota modulation in older adults.

### 5.2 Resistance exercise

Resistance exercise refers to physical exercise that involves applying external loads (e.g., dumbbells, resistance bands, or machines) to stimulate muscle contraction, thereby enhancing muscle strength and muscle mass. Numerous studies have demonstrated that resistance exercise can increase the cross-sectional area of both type I and type II muscle fibers, promote muscle protein synthesis (MPS), and stimulate muscle hypertrophy (Sayer et al., 2024). Consequently, it has been widely recognized as an effective intervention for the prevention and treatment of sarcopenia in older adults.

However, current evidence regarding the impact of resistance exercise on SCFA levels in older populations remains limited and inconclusive. McKenna et al. (2021) reported that a 10-week resistance exercise program (three sessions per week at 75% 1 repetition maximum) significantly increased the relative abundance of SCFA-producing taxa such as *Veillonellaceae* and *Akkermansia* in the gut microbiota of overweight middle-aged and older adults (50–64 years), alongside notable improvements in upper and lower limb muscle strength. In contrast, Agyin-Birikorang et al. (2025) observed that a 10-week resistance exercise intervention (twice weekly at 60% 1 repetition maximum) in previously untrained healthy older individuals (60–80 years) improved skeletal muscle mass but had no significant effects on gut microbial *α*- or *β*-diversity, nor did it substantially alter fecal or serum SCFA concentrations. These inconsistencies may stem from differences in baseline gut microbiota composition, dietary control, and variations in training intensity and frequency among study populations (Van Hul and Cani, 2023). Additionally, individual differences in age, metabolic status, and baseline muscle fitness may also affect the effects of resistance exercise on gut microbiota and its metabolites (Wen and Duffy, 2017).

Therefore, the specific effects of resistance exercise on SCFA-producing bacteria and SCFA levels in older adults remain to be fully elucidated. Future studies using standardized protocols, larger sample sizes, and longitudinal designs are needed to determine whether resistance exercise can induce microbial-derived SCFAs production.

### 5.3 Combined exercise

Combined exercise, referring to the integration of aerobic and resistance training within a single intervention program, is widely recognized as an effective strategy to synergistically harness the respective benefits of both modalities. By simultaneously improving cardiovascular endurance and increasing muscle strength and mass, combined exercise may provide more comprehensive health benefits, particularly in older adults (Villareal et al., 2017). In recent years, the effects of combined exercise on gut SCFA-producing bacteria and SCFA levels in older adults have attracted growing research interest. Zhong et al. (2021) demonstrated that an 8-week combined exercise intervention significantly increased the relative abundance of SCFA-producing taxa—including *Bifidobacteriaceae*, *Lachnospiraceae*, and *Mitsuokella*—in sedentary older women, while concurrently reducing pro-inflammatory bacteria such as *Proteobacteria*. Similarly, Erlandson et al. (2021) reported that a 24-week combined exercise program in older adults was associated with increased abundances of *Bifidobacterium*, *Oscillospira*, and *Anaerostipes*, along with elevated fecal butyrate levels. These findings suggest that combined exercise may promote the enrichment of SCFA-producing bacteria in the gut of older adults, thereby increasing SCFA production.

Notably, compared with aerobic or resistance exercise alone, combined exercise appears to be more effective at stimulating SCFA production. For example, Ma et al. (2020) compared the effects of 8-week aerobic, resistance, and combined exercise interventions on serum SCFA levels in db/db mice. They found that although all exercise modalities significantly increased serum SCFA concentrations compared to controls, the combined exercise group showed higher acetate and butyrate levels than the aerobic group, and higher propionate and valerate levels than the resistance group, indicating a superior effect of combined exercise on SCFA production (Ma et al., 2020). The superior SCFA-producing effects of combined exercise may be attributed to the complementary and synergistic physiological mechanisms of the two exercise types. Aerobic exercise primarily enhances gastrointestinal motility and improves mucosal blood flow, thereby creating a gut environment favorable for SCFA-producing anaerobes (Huang et al., 2024). Meanwhile, resistance exercise produces large amounts of lactate through anaerobic glycolysis. Lactate can serve as a substrate for certain gut bacteria (e.g., *Veillonella*), which convert it into propionate and other SCFAs, further increasing SCFA levels (Zhao et al., 2019; Zhang and Huang, 2023).

In summary, combined exercise, which integrates the advantages of both aerobic and resistance exercise, synergistically promotes the proliferation of SCFA-producing bacteria in the gut and may represent the most effective strategy for enhancing SCFA levels. However, high-quality clinical studies specifically targeting older adults are lacking, and the precise effects of different exercise modalities on SCFA levels in this population remain to be fully elucidated. Future research should also refine parameters such as exercise intensity, frequency, and duration to develop optimized and personalized exercise prescriptions aimed at increasing SCFA levels in older individuals.

## 6 Mechanisms of exercise-induced SCFAs in the prevention and treatment of sarcopenia

**Figure 1:**
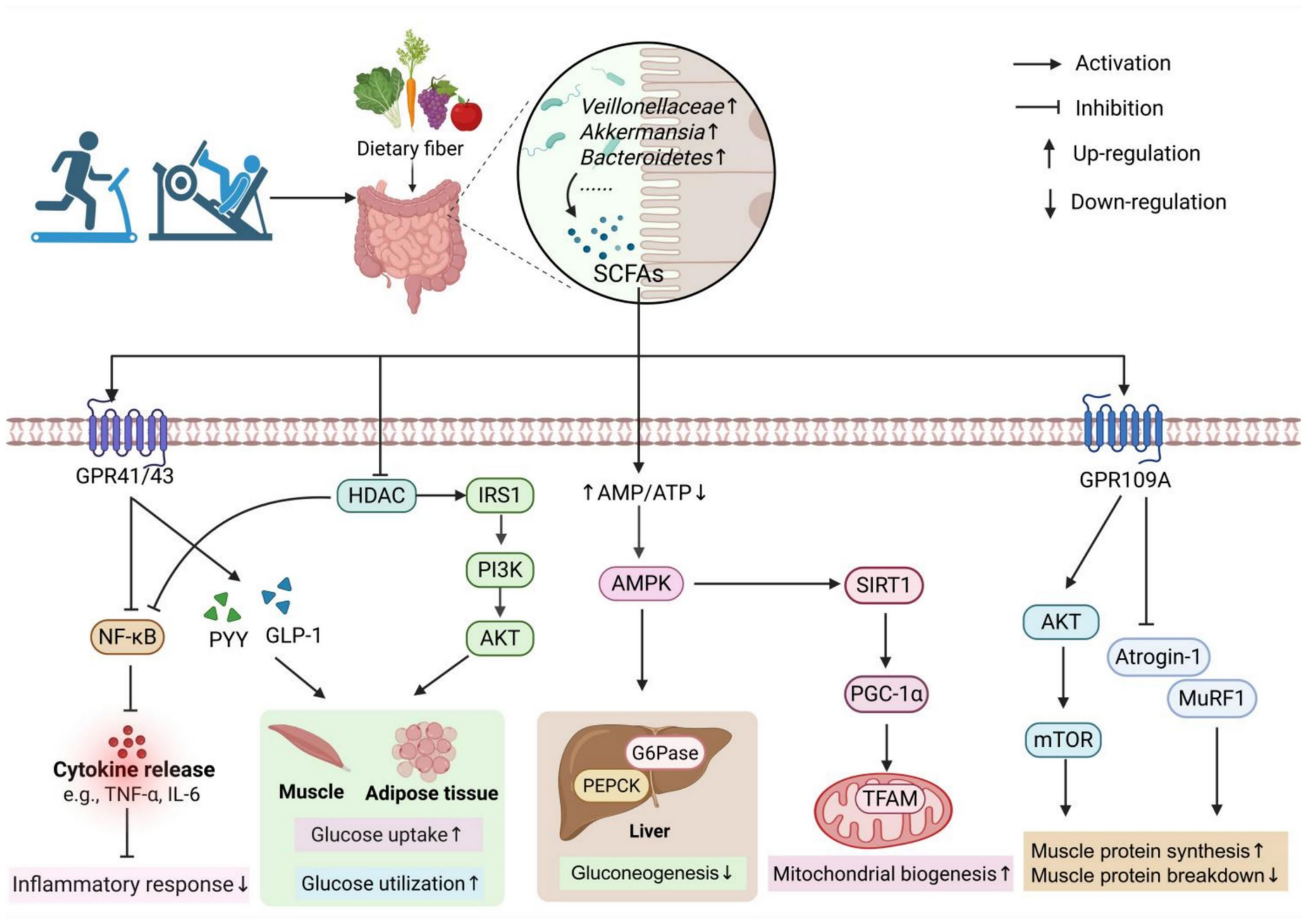
The mechanism of exercise-induced SCFAs in the prevention and treatment of sarcopenia. Exercise can increase the abundance of SCFA-producing bacteria in the gut, thereby promoting SCFA production. SCFAs exert their effects by interacting with GPR41/GPR43 receptors or by passively diffusing into cells to inhibit HDAC activity, which in turn suppresses the NF-κB pathway and reduces inflammatory responses. SCFAs also stimulate the secretion of PYY and GLP-1 by activating GPR41/GPR43 receptors on enteroendocrine cells in the colon. In addition, SCFAs can activate the IRS1/PI3K/AKT pathway through HDAC inhibition, thereby enhancing glucose uptake and utilization in skeletal muscle and adipose tissue. Moreover, SCFAs can increase the AMP/ATP ratio and activate the AMPK pathway, leading to reduced hepatic gluconeogenesis. SCFAs can also activate GPR109A on muscle cell membranes, which subsequently activates the AKT/mTOR pathway and inhibits the expression of MuRF1 and Atrogin-1. These actions collectively promote muscle protein synthesis, suppress protein degradation, and ultimately alleviate muscle atrophy. The figure was created using BioRender.com.

Exercise-induced increases in SCFA levels may play a pivotal role in mediating the beneficial effects of physical activity on skeletal muscle health in older adults. SCFAs act as important signaling molecules that influence various physiological processes, including muscle protein turnover, inflammatory regulation, mitochondrial homeostasis, and metabolic function. In this section, we summarize the potential mechanisms through which exercise-induced SCFAs contribute to the maintenance of muscle mass and function, providing novel insights into their therapeutic implications for age-related muscle loss (Figure 1).

### 6.1 Improving muscle protein metabolism

Skeletal muscle mass are largely maintained by a dynamic balance between protein synthesis and degradation. Among these processes, the mTORC pathway is a key regulator of muscle protein synthesis. Activation of mTORC1 promotes the phosphorylation of downstream targets such as ribosomal protein S6 kinase (p70S6K) and inhibits eukaryotic translation initiation factor 4E-binding protein 1 (4EBP1), thereby facilitating protein synthesis and muscle hypertrophy (Bodine et al., 2001).

Conversely, muscle protein degradation is mainly mediated by two catabolic systems: the ubiquitin–proteasome system (UPS) and the autophagy–lysosomal pathway. Both are tightly regulated by forkhead box O (FoxO) family transcription factors. Aging is associated with increased inflammation and oxidative stress, leading to reduced protein kinase B (AKT) activity. Reduced AKT signaling promotes the nuclear translocation of dephosphorylated FoxO, which upregulates the expression of key autophagy-related genes (e.g., LC3, Atg4, Beclin1), accelerating protein breakdown and contributing to muscle atrophy (Cui et al., 2020). Additionally, FoxO can activate UPS by inducing the expression of E3 ubiquitin ligases such as muscle ring finger protein-1 (MuRF1) and muscle atrophy F-box protein (Atrogin-1), further promoting muscle protein degradation (Tang et al., 2014).

Evidence suggests that age-related declines in SCFA levels disrupt the anabolic–catabolic balance of muscle protein, favoring increased degradation and reduced synthesis, ultimately leading to sarcopenia (Jiang et al., 2022). However, restoring SCFA levels through dietary strategies or probiotic supplementation reverses these trends, indicating that SCFAs play a crucial role in regulating muscle protein metabolism (Chen et al., 2021; She et al., 2025).

SCFAs exert their effects on protein metabolism mainly through two interrelated mechanisms: activating the mTOR pathway and inhibiting the FoxO-mediated degradation pathway. For example, Mo et al. (2025) found that in mice with sarcopenic obesity induced by a high-fat diet, melatonin supplementation modulated the gut microbiota, promoted SCFA production, and subsequently activated the mTOR/p70S6K signaling pathway, leading to improved muscle mass and strength. Similarly, Liu et al. (2024) reported that exogenous supplementation with SCFAs (acetate, propionate, and butyrate) activated the mTOR/S6K1 pathway in the skeletal muscle of aged SAMP8 mice, alleviating age-related muscle atrophy and functional decline.

*In vitro* studies further support these findings. SCFAs, particularly butyrate, have been shown to promote myotube growth by inhibiting the FoxO3a/Atrogin-1 pathway and activating the mTOR signaling cascade, thereby reducing muscle protein catabolism and increasing muscle protein synthesis, respectively (Liu et al., 2024). In dexamethasone-induced atrophy models, butyrate decreased the gene expression of Atrogin-1 and MuRF1, and enhanced AKT/mTOR signaling, thereby attenuating muscle atrophy (Zhao et al., 2025). Notably, the protective effects of butyrate were diminished when GPR109A was inhibited using pertussis toxin, indicating that SCFA-mediated muscle protection is at least partially dependent on GPR109A signaling (Zhao et al., 2025).

Beyond direct intracellular signaling, SCFAs also enhance muscle protein anabolism by improving nutrient absorption. Probiotic supplementation following endurance training has been shown to increase the abundance of SCFA-producing bacteria, such as *Bacteroides* and *Prevotella*, upregulate intestinal tight junction proteins, and activate gastric protein synthesis pathways (Lamprecht et al., 2012). These changes improve gut barrier integrity, enhance amino acid absorption efficiency, and promote systemic nutrient availability for muscle protein synthesis (Lamprecht et al., 2012). Similarly, a comparative study found that professional rugby players exhibited higher SCFA levels than sedentary controls, which were associated with increased ATP production, enhanced dietary protein utilization, and greater skeletal muscle protein synthesis (Barton et al., 2018). While these observations suggest that microbiota-derived SCFAs may promote muscle protein anabolism by improved nutrient absorption, important limitations remain: SCFAs were not directly measured in the probiotic study, the rugby player study assessed stool rather than circulating levels, and differences in diet quality or protein intake may also explain the associations. Thus, although these studies provide intriguing mechanistic insights, the causal contribution of SCFAs to skeletal muscle anabolism in humans remains to be firmly established and requires trials with direct SCFA measurements and controlled dietary assessments.

In summary, SCFAs may improve muscle protein metabolism and help prevent age-related sarcopenia through multiple mechanisms, including activating anabolic pathways (e.g., mTOR signaling), suppressing catabolic processes (e.g., FoxO signaling), and enhancing nutrient absorption by improving gut barrier function. However, it should be noted that most available evidence derives from studies employing exogenous SCFAs, while human data are limited to indirect associations (e.g., higher dietary fiber intake linked to greater muscle strength) (Otsuka et al., 2023), and no interventional studies have yet directly demonstrated effects on muscle protein turnover. Whether exercise-induced SCFAs exert similar beneficial effects and share the same underlying mechanisms on muscle protein metabolism remains to be fully elucidated and warrants further investigation.

### 6.2 Reduced of inflammatory response

Aging is associated with a chronic, low-grade systemic inflammatory state, known as “inflammaging,” which is characterized by persistently elevated circulating levels of cytokines such as tumor necrosis factor-*α* (TNF-α), interleukin-1β (IL-1β), and interleukin-6 (IL-6) (Crunkhorn, 2020). A vicious cycle between augmented pro-inflammatory signaling and cellular senescence further amplifies inflammaging, contributing to the development of several age-related disorders, including sarcopenia (Wang, 2022).

One of the central mechanisms driving inflammaging is gut microbiota dysbiosis (Xu et al., 2024). With advancing age, there is a marked decline in beneficial SCFA-producing bacteria, including *Lactobacillus*, *Bacteroides*, and *Faecalibacterium prausnitzii*, accompanied by an overgrowth of Gram-negative, LPS-producing bacteria (O'Toole and Jeffery, 2015). This microbial shift not only reduces SCFA production but also elevates LPS levels, thereby compromising gut barrier integrity (Yu et al., 2024). Consequently, the reduction in anti-inflammatory capacity, together with increased systemic LPS levels, promotes skeletal muscle inflammation. Indeed, circulating LPS can activate Toll-like receptor 4 (TLR4) on muscle cell membranes, triggering the nuclear factor-kappa B (NF-κB) signaling cascade and promoting the expression of pro-inflammatory cytokines, such as TNF-α and IL-1β (Sailaja et al., 2022). These cytokines impair protein homeostasis by inhibiting the Akt/mTOR signaling pathway, while simultaneously activating catabolic pathways, particularly the UPS and autophagy–lysosome systems, ultimately leading to increased muscle protein degradation and muscle atrophy (Draganidis et al., 2016). Therefore, an inflammatory response induced by SCFA deficiency may represent a key contributing factor in the pathogenesis of sarcopenia.

Emerging evidence suggests that exercise exerts its anti-inflammatory effects, in part, through the modulation of gut microbiota composition and the consequent enhancement of SCFA production. Vijay et al. (2021) reported that a 6-week exercise intervention in community-dwelling older adults significantly increased serum butyrate levels, which were inversely correlated with circulating concentrations of TNF-α and IL-6. Huang et al. (2022) found that 12 weeks of aerobic exercise in apolipoprotein E (APOE) knockout mice markedly increased the fecal abundance of SCFA-producing genera such as *Rikenellaceae* and *Dubosiella*, accompanied by elevated SCFA levels and decreased expression of TNF-α and IL-1β.

Mechanistically, SCFAs can directly bind to GPRs on the membranes of immune and epithelial cells or passively diffuse into cells to inhibit histone deacetylases (HDACs) activity, resulting in epigenetic modifications that suppress the NF-κB signaling pathway (Evans et al., 2020). For example, exogenous supplementation with acetate, propionate, and butyrate has been shown to attenuate inflammatory responses by modulating GPR43/HDAC3 signaling and inhibiting the LPS/TLR4/NF-κB pathway in mice with high-fat diet-induced metabolic injury (Wang et al., 2024). Similarly, in LPS-pretreated neutrophils, butyrate and propionate suppress NF-κB activation by inhibiting HDAC activity (Aoyama et al., 2010). Furthermore, Li et al. (2020) reported that in hypercholesterolemic mice, voluntary wheel running not only increased the relative abundances of *Lactobacillus* and *Eubacterium nodatum* but also upregulated colonic mRNA expression of GPR109A and GPR41, enhanced SCFA levels (e.g., acetate, propionate, and isobutyrate), and concurrently downregulated inflammatory markers. These findings suggest that exercise-induced SCFAs may exert anti-inflammatory effects through the activation of GPRs and the inhibition of HDACs, thereby suppressing the NF-κB signaling pathway.

Moreover, SCFAs enhance intestinal barrier integrity, thereby indirectly reducing systemic inflammation. Specifically, SCFAs increase transepithelial electrical resistance and upregulate the expression of tight junction proteins such as zonula occludens-1 (ZO-1) and occludin, thereby strengthening gut barrier function (Mann et al., 2024). This prevents the translocation of bacterial toxins (e.g., LPS) into the bloodstream, effectively reducing upstream triggers of skeletal muscle inflammation and ultimately mitigating inflammatory damage (Roy et al., 2025).

In summary, exercise-induced SCFAs may attenuate chronic low-grade systemic inflammation by activating GPRs signaling, inhibiting HDAC activity, strengthening gut barrier integrity, and reducing LPS translocation, thereby collectively suppressing NF-κB signaling pathways. Therefore, in older adults, exercise-induced SCFAs may play a critical role in preventing and managing age-related sarcopenia through anti-inflammaging mechanisms.

### 6.3 Improvement of insulin resistance

The incidence of insulin resistance increases with age (Walker et al., 2021). With advancing age, abnormal accumulation of intermuscular fat and intramyocellular lipid (IMCL), along with elevated levels of pro-inflammatory cytokines, reduces insulin sensitivity in skeletal muscle, liver, and other peripheral tissues (Chee et al., 2016). This decline in insulin responsiveness impairs glucose homeostasis and ultimately leads to systemic insulin resistance. On one hand, insulin resistance downregulates insulin-like growth factor-1 (IGF-1) signaling and inhibits the downstream phosphoinositide 3-kinase (PI3K)/Akt/mTOR pathway, resulting in reduced glucose uptake and utilization and consequently diminishing skeletal muscle protein synthesis (Abdul-Ghani and DeFronzo, 2010; Ji et al., 2025). On the other hand, insulin resistance activates the PI3K/Akt/FoxO pathway, thereby upregulating the expression of muscle atrophy-related genes, such as Atrogin-1 and MuRF-1 (Kitajima et al., 2020; Renna et al., 2019). This promotes skeletal muscle protein degradation and ultimately leads to muscle atrophy. Therefore, insulin resistance is considered an important pathogenic mechanism contributing to the development of sarcopenia.

A large body of research has demonstrated that SCFAs are key modulators involved in regulating insulin sensitivity. SCFAs bind to G-protein coupled receptors GPR43 and GPR41 on the membranes of colonic enteroendocrine L cells, stimulating the secretion of peptide YY (PYY), which enhances glucose uptake and utilization in skeletal muscle and adipose tissue (Boey et al., 2006). Further, SCFAs activate GPR43 on colonic cells, promoting the release of glucagon-like peptide-1 (GLP-1) (Kimura et al., 2013). GLP-1 binds to GLP-1 receptors on pancreatic *β* and *δ* cells, thereby enhancing insulin secretion, suppressing glucagon secretion, and ultimately maintaining blood glucose homeostasis (Drucker, 2018).

In addition, SCFAs can increase the adenosine monophosphate (AMP) to adenosine triphosphate (ATP) ratio, thereby activating the AMP-activated protein kinase (AMPK) signaling pathway (Tao and Wang, 2025). In skeletal muscle, SCFA-induced AMPK activation has been shown to enhance mitochondrial fatty acid oxidation, increase energy expenditure, and improve insulin sensitivity in mice (Gao et al., 2009). In the liver, AMPK activation suppresses the expression of gluconeogenic enzymes such as glucose-6-phosphatase (G6Pase) and phosphoenolpyruvate carboxykinase (PEPCK), thereby inhibiting gluconeogenesis (Canfora et al., 2015). Indeed, dietary acetate supplementation has been shown to upregulate hepatic AMPK expression and downregulate gluconeogenesis-related gene expression in diabetic mice, leading to reduced blood glucose levels (Sakakibara et al., 2006). These findings suggest that SCFAs inhibit hepatic gluconeogenesis and enhance peripheral glucose utilization by activating the AMPK pathway, thereby improving insulin resistance.

Moreover, SCFAs can enhance skeletal muscle insulin sensitivity through epigenetic modifications. HDAC play a crucial role in chromatin structure remodeling and gene expression regulation by deacetylating histones, thus controlling chromatin accessibility and transcriptional activity (Cheng et al., 2024). Chriett et al. (2017) found that in L6 myotubes with palmitate-induced insulin resistance, butyrate increased histone H3 acetylation near the insulin receptor substrate 1 (IRS1) gene promoter, significantly upregulating IRS1 mRNA and protein expression levels. IRS1, a key initiator of insulin signaling, is mainly distributed in peripheral tissues such as skeletal muscle. Upon activation, IRS1 interacts with the p85 regulatory subunit of PI3K, subsequently activating AKT and promoting the translocation of glucose transporter type 4 (GLUT4), ultimately enhancing glucose uptake and utilization in skeletal muscle and adipose tissue (Maarbjerg et al., 2011; Bo et al., 2024). Furthermore, acetate injection in diabetic rats has been shown to significantly increase GLUT4 mRNA and protein expression levels in skeletal muscle (Yamashita et al., 2009).

Collectively, SCFAs play a pivotal role in improving insulin resistance through multiple mechanisms, including activating GPR41/43-mediated intestinal hormone release, enhancing AMPK-mediated metabolic regulation, and modulating epigenetic modifications to upregulate key insulin signaling molecules such as IRS1 and PI3K/AKT/GLUT4. Therefore, exercise-induced increases in SCFA production may help ameliorate age-related insulin resistance, restore muscle protein synthesis, and reduce protein degradation, ultimately preventing or mitigating sarcopenia in older adults.

### 6.4 Improve mitochondrial dysfunction

Mitochondrial function gradually decreases with age (Harrington et al., 2023). Studies have shown that the number of butyrate producing bacteria in the gut of the elderly is significantly reduced, which leads to the decrease of antioxidant capacity, the increase of reactive oxygen species (ROS) level, and the induction of oxidative damage to mitochondrial DNA (mtDNA), ultimately resulting in mitochondrial dysfunction (Pichaud et al., 2019). Additionally, excessive ROS in skeletal muscle not only upregulates the expression of myostatin, Atrogin-1, and MuRF-1, but also modulates key transcription factors such as NF-κB and FoxO (Ferri et al., 2020). These molecular changes activate both the UPS and the autophagy–lysosomal pathway, leading to impaired mitochondrial function, increased apoptosis, and defective autophagy, thereby contributing to skeletal muscle atrophy (Phua et al., 2024; Guo et al., 2023).

In addition, peroxisome proliferator-activated receptor *γ* coactivator 1-alpha (PGC-1α) is a crucial regulator of mitochondrial function. Upon activation by AMPK phosphorylation and silent information regulator 1 (SIRT1) deacetylation, PGC-1α interacts with downstream molecules such as nuclear factor erythroid 2-related factor 1 (NRF1) and mitochondrial transcription factor A (TFAM), promoting mitochondrial biogenesis and maintaining mitochondrial homeostasis (Herzig and Shaw, 2018). However, during aging, the PGC-1α–NRF1–TFAM signaling pathway in skeletal muscle is impaired, resulting in reduced mitochondrial number and function, which subsequently contributes to sarcopenia (Affourtit and Carré, 2024). Therefore, improving mitochondrial function in skeletal muscle help delay the onset and progression of sarcopenia.

It is well established that exercise acts as an effective activator of mitochondrial function. Studies have shown that aerobic exercise upregulates the expression of PGC-1α, AMPK, and SIRT1 in skeletal muscle, thereby activating downstream effectors such as Nrf2 and TFAM (Smith et al., 2023). This cascade promotes mitochondrial biogenesis, enhances oxidative metabolic capacity, and mitigates muscle loss (Gan et al., 2018). Additionally, resistance exercise has been shown to improve mitochondrial function, increase mitochondrial density and enzymatic activity, and consequently enhance muscle mass and strength, thereby delaying age-related muscle atrophy (Lei et al., 2024). Recently, Uchida et al. (2023) reported that endurance exercise selectively modulates the gut microbiota, increases the abundance of SCFA-producing bacteria, and elevates skeletal muscle endurance, citrate synthase activity, and PGC-1α levels. Interestingly, when mice were treated with antibiotics, these exercise-induced mitochondrial adaptations were markedly diminished, suggesting that improvements in mitochondrial function mediated by exercise are closely linked to an increase in SCFA-producing bacteria (Uchida et al., 2023).

In fact, both animal and cell studies have demonstrated that SCFAs are key mediators in regulating skeletal muscle mitochondrial energy metabolism. Walsh et al. (2015) found that butyrate attenuated muscle atrophy in aged mice by inhibiting HDAC expression in skeletal muscle, which led to upregulation of mitochondrial porin and TFAM levels, and enhanced oxidative metabolic capacity. In a high-fat diet-induced obese mouse model, butyrate supplementation significantly increased the expression of PGC-1α and AMPK in skeletal muscle, accompanied by improved mitochondrial function and biogenesis, indicating that butyrate promotes mitochondrial biogenesis via activation of the PGC-1α/AMPK signaling pathway (Hong et al., 2016). Similarly, Maruta et al. (2016) observed in L6 myotubes that acetate treatment induced AMPK phosphorylation and upregulated both gene and protein expression of GLUT4 and myoglobin, thereby improving lipid metabolism in skeletal muscle.

In conclusion, exercise-induced SCFAs may activate the PGC-1α/AMPK/TFAM signaling pathway by inhibiting HDAC activity, thereby improving mitochondrial function in skeletal muscle and ultimately delaying the onset and progression of sarcopenia. However, direct evidence demonstrating that exercise-induced changes in gut microbiota-derived SCFAs improve skeletal muscle mitochondrial function and prevent sarcopenia remains limited. Therefore, further studies are needed to confirm this mechanism.

## 7 Summary and perspective

In conclusion, SCFAs may serve as critical mediators underlying the preventive and therapeutic effects of exercise on sarcopenia. Current evidence indicates that various exercise modalities, particularly combined exercise, can significantly increase the relative abundance of SCFA-producing bacteria in the gut of older adults, thereby enhancing SCFA production. Moreover, SCFAs may exert their effects through mechanisms such as activating GPR41/43 signaling pathways, inhibiting HDAC activity, and improving intestinal barrier function, thereby promoting muscle protein synthesis, suppressing inflammatory responses, enhancing mitochondrial function, and improving insulin sensitivity, ultimately contributing to the attenuation of age-related muscle atrophy.

However, it is important to note that most human studies to date have not directly assessed SCFA levels, and evidence regarding certain exercise modalities (particularly resistance exercise) remains limited. Additionally, the specific molecular mechanisms through which exercise promotes SCFA production and mitigates sarcopenia are largely based on indirect and fragmented evidence and require further validation. It also remains unclear which exercise intensity, duration, and frequency are most effective in enhancing SCFA-producing bacteria and systemic SCFA levels in older adults. Furthermore, potential safety concerns and exercise tolerance in this population should be carefully considered when designing interventions. Therefore, determining the optimal individualized exercise prescription to maximize improvements in skeletal muscle mass and function among older adults represents an important direction for future research.

Moreover, although preclinical studies have shown that butyrate can modulate muscle-related pathways—for instance, by engaging GPR109A-dependent signaling or enhancing IRS1 expression in myotube models—in humans, butyrate undergoes extensive first-pass metabolism in the gut and liver and reaches peripheral skeletal muscle at negligible levels. These findings should therefore be viewed as mechanistic evidence rather than direct proof of *in vivo* effects in older adults. Future human studies with direct measurements of circulating SCFAs and skeletal-muscle endpoints are needed to establish the extent to which these mechanisms operate in vivo.

In addition to SCFAs, other nutritional factors, such as dietary protein intake and the bioavailability of essential amino acids (EAAs), also play indispensable roles in regulating the balance of muscle protein synthesis and degradation in response to exercise (Deane et al., 2021; Ely et al., 2023). Recent studies suggest that combining exercise with dietary modifications (e.g., adequate protein/dietary fiber intake) or probiotic supplementation may exert synergistic effects on gut microbiota modulation and SCFA production, thereby offering additional benefits for muscle health (Sun et al., 2022; Moura et al., 2024; Lin et al., 2023; Moreno-Pérez et al., 2018). While the present review has focused on the potential role of exercise-induced SCFAs in skeletal muscle protein metabolism, future studies should adopt integrated approaches that incorporate these additional mechanisms to provide safer, more effective, and more comprehensive strategies for the prevention and treatment of age-related sarcopenia.
